# Beyond the Fragmentation Threshold Hypothesis: Regime Shifts in Biodiversity Across Fragmented Landscapes

**DOI:** 10.1371/journal.pone.0013666

**Published:** 2010-10-27

**Authors:** Renata Pardini, Adriana de Arruda Bueno, Toby A. Gardner, Paulo Inácio Prado, Jean Paul Metzger

**Affiliations:** 1 Departamento de Zoologia, Instituto de Biociências, Universidade de São Paulo, São Paulo, Brazil; 2 Department of Zoology, University of Cambridge, Cambridge, United Kingdom; 3 Departamento de Ecologia, Instituto de Biociências, Universidade de São Paulo, São Paulo, Brazil; University of Western Ontario, Canada

## Abstract

Ecological systems are vulnerable to irreversible change when key system properties are pushed over thresholds, resulting in the loss of resilience and the precipitation of a regime shift. Perhaps the most important of such properties in human-modified landscapes is the total amount of remnant native vegetation. In a seminal study Andrén proposed the existence of a fragmentation threshold in the total amount of remnant vegetation, below which landscape-scale connectivity is eroded and local species richness and abundance become dependent on patch size. Despite the fact that species patch-area effects have been a mainstay of conservation science there has yet to be a robust empirical evaluation of this hypothesis. Here we present and test a new conceptual model describing the mechanisms and consequences of biodiversity change in fragmented landscapes, identifying the fragmentation threshold as a first step in a positive feedback mechanism that has the capacity to impair ecological resilience, and drive a regime shift in biodiversity. The model considers that local extinction risk is defined by patch size, and immigration rates by landscape vegetation cover, and that the recovery from local species losses depends upon the landscape species pool. Using a unique dataset on the distribution of non-volant small mammals across replicate landscapes in the Atlantic forest of Brazil, we found strong evidence for our model predictions - that patch-area effects are evident only at intermediate levels of total forest cover, where landscape diversity is still high and opportunities for enhancing biodiversity through local management are greatest. Furthermore, high levels of forest loss can push native biota through an extinction filter, and result in the abrupt, landscape-wide loss of forest-specialist taxa, ecological resilience and management effectiveness. The proposed model links hitherto distinct theoretical approaches within a single framework, providing a powerful tool for analysing the potential effectiveness of management interventions.

## Introduction

Regime shifts represent fundamental, sudden changes in ecosystem state, and are usually driven by changes to key-variables that are linked to ecological resilience – the capacity of the system to absorb disturbance and reorganize so as to retain essentially the same function, structure, identity and feedbacks [1]. Multiple stable-states have been experimentally demonstrated in a variety of ecological systems [2], and accumulating evidence suggests that regime shifts can occur in a number of complex ecosystems, with potentially catastrophic consequences to biodiversity, ecosystem services and human well-being [3], [4].

The ability to anticipate such dramatic changes is of foremost importance to ecosystem management and has inspired a growing amount of research and modeling work [5]–[7]. However, our understanding of the mechanisms that may underpin regime shifts is largely limited to aquatic environments and semi-arid rangelands [4], [8]. While there is growing support for the notion that human-driven impacts can induce sudden changes in other ecosystems, including tropical forests [9], [10], the definition of the key variables and feedbacks governing such shifts remain one of the greatest challenges facing the management of human-modified landscapes especially in the tropics [11].

Candidate drivers of potentially irreversible ecological shifts in human-modified landscapes include changes in (1) the total amount and configuration of native vegetation cover through its effects on landscape connectivity, (2) vegetation structure through its influence on natural disturbance regimes, and (3) species composition and the potential for ecological cascades [12]. Among these options, the total amount of native vegetation cover has been found to be fundamentally important for all major aspects of landscape management [13], with a growing amount of empirical evidence linking changes in total vegetation cover to changes in both biodiversity [14], [15] and ecosystem function [16], [17].

The relevance of total native vegetation cover as a driver of ecological change in fragmented landscapes was initially suggested by simulation studies. This body of work has shown that total habitat cover is non-linearly related to both patch (e.g. number of patches, size of the largest patch, and landscape percolation) and gap (e.g. mean distance to the nearest patch, and lacunarity, i.e. variability in gap size) structure in fragmented landscapes [18], [19], and thus should govern thresholds in dispersal success [18], landscape connectivity [20], and overall persistence of individual species (“extinction thresholds”) [21], [22]. Moreover, although simulated landscapes differ from real landscapes, with their complex array of socio-economic and biophysical constraints, general trends in landscape structure changes tend to remain similar in real landscapes [23]. Nevertheless, these simulation studies contrast markedly with the majority of empirical work during the same period, which focussed largely on island biogeography theory and on the patch, rather the landscape scale [19].

In a seminal meta-analysis study Andrén [24] (see also [25]) proposed the existence of a fragmentation threshold in the total amount of remnant vegetation, below which landscape connectivity is eroded (i.e. the landscape is broken into several isolated patches) and local species abundance and richness (alpha diversity) become dependent on the size or isolation of remaining patches. Notably, this threshold links landscape-context and patch-area effects, drawing on both theoretical evidence for structural thresholds in landscape pattern [18]–[20] and the known importance of patch size and isolation as determinants of local extinction risk [26]–[28].

Nevertheless, despite the fact that species patch-area effects have been a mainstay of conservation science for more than three decades there has yet to be a robust empirical evaluation of the species patch-area relationship across multiple landscapes that differ in the amount of total vegetation cover. Few empirical studies have directly addressed the consequence of changes in total native vegetation cover for multiple species [12] (but see [14], [29]), and those that considered both landscape and patch-scale variables are confounded by inappropriate designs and uncontrolled variables [30]. Moreover, the relevance of such a threshold for guiding the management of fragmented landscapes has been questioned [13], [31], [32]. Since responses are expected to vary among species depending on habitat preferences and dispersal capacity [33], relatively minor changes in native vegetation cover are not expected to cause abrupt changes in biodiversity across fragmented landscapes [12].

Here we expand Andrén's hypothesis, and propose that the fragmentation threshold represents a first step in a positive feedback mechanism that has the capacity to severely impair ecological resilience, and drive a potentially irreversible regime shift in biodiversity of fragmented landscapes. We present a new conceptual model that makes explicit the key variables and feedbacks governing such a shift, taking into account the interaction between native vegetation cover at patch and landscape scales, and between alpha (patch) and gamma (landscape) diversity. The model considers that: (1) population sizes and local extinction risk are determined by patch size, while immigration rates are determined by landscape-scale vegetation cover, and (2) the capacity to recover from local species losses (reduction in alpha diversity) is dependent on the total landscape species pool (gamma diversity). From the model, we derive general predictions of how species abundance and diversity patterns at different spatial scales should change along a gradient of native vegetation loss, and test these predictions using a dataset on the distribution of non-volant small mammals across replicate landscapes in the Atlantic forest of Brazil.

Our results were consistent with the proposed model. The species-area relationship among forest patches was strongly dependent upon the total amount of remaining forest and was only observed at an intermediate level of forest cover (where gamma diversity was high but alpha diversity was dependent on patch size). At a high level of forest cover both gamma and alpha diversity were high, whereas a low level of forest cover was associated with a homogeneously low alpha diversity, and thus an abrupt drop in gamma diversity and associated ecological resilience. The regime-shift model presented here provides a powerful analytical and diagnostic framework for understanding the potential effectiveness of management interventions, and guiding the investment of limited conservation resources in human-modified ecosystems.

## Materials and Methods

### Ethics Statement

Trapping and handling were approved by IBAMA - Instituto Brasileiro do Meio Ambiente e dos Recursos Naturais Renováveis (permissions 57/02 - IBAMA/SP, 11/04 - IBAMA/SP, 168/2004 - CGFAU/LIC, 237/2005 - CGFAU/LIC, and 262/2006 – COFAN) and conformed to guidelines sanctioned by the American Society of Mammalogists Animal Care and Use Committee. Because our study involved only the capture, handling for marking and the immediate release of small rodents and marsupials in the field, it did not receive an approval from the Ethics Committee of the Institute of Biosciences - University of São Paulo (Comissão de Ética em Uso de Animais Vertebrados em Experimentação – CEA - http://ib.usp.br/etica_animais.htm), which only requires approval for studies on vertebrates that include experimentation (e.g. maintenance in captivity, injection of drugs, or surgery).

### The Conceptual Model

**Figure 1:**
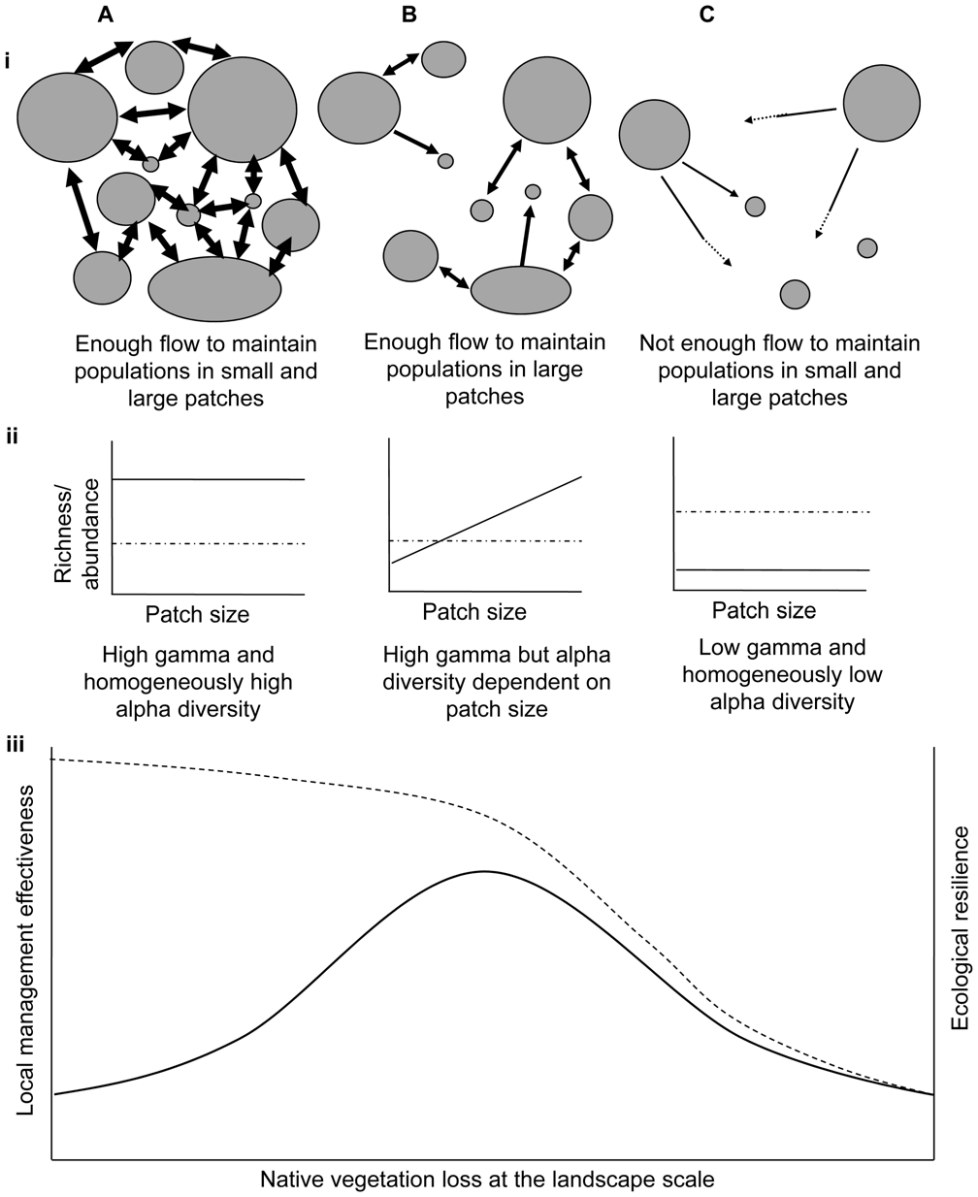
Regime shift in biodiversity along a gradient of increasing native vegetation loss (A, B, C). i. Schematic representation of the distribution of native vegetation patches and landscape immigration rates (proportionate to width of arrows). ii. Expected relationship between patch size and richness or abundance (per unit of area) of specialist (solid line) and generalist (dotted line) species. iii. Expected relationship between both the effectiveness of local management (solid line) and ecological resilience of native biota (dotted line) to landscape-wide native vegetation loss.

Our conceptual model assumes that the distribution and abundance of habitat specialist species (i.e. those that are known from independent work to depend on native vegetation) in fragmented landscapes is mediated by two key factors: local resource availability (determined essentially by patch size), and landscape immigration rates (determined by the connectivity amongst inhabitable vegetation patches). The loss of native vegetation is postulated to decrease ecological resilience – defined here as the capacity of the landscape-wide biota to recover from local species losses in individual patches – through reduced immigration at the landscape scale. Where native vegetation cover is high, immigration rates are high across the landscape because of the close proximity among patches, allowing for quick recovery from local species losses (i.e. high ecological resilience). Thus densities of species and individuals of each species are comparatively high throughout the landscape, irrespective of differences in the size of individual patches (Figure 1A). As the loss of native vegetation proceeds, connectivity among patches decreases to the point that the persistence of individual species within a given patch becomes dependent on the size of the patch [24], since reduced immigration rates are insufficient to maintain smaller populations, which in turn are more vulnerable to local extinction from stochastic events. Although some species are restricted to larger patches, the regional pool of species is maintained and ecological resilience is not severely impaired (Figure 1B). At this point, the loss of species from smaller patches further increases the isolation of remaining populations across the landscape, making species persistence in the still occupied larger patches also vulnerable to further losses of native vegetation at the landscape scale. At the same time small additional losses of native vegetation at increasingly low levels of total cover (∼10–20%) result in an exponential increase in the mean and variance of distances among patches [18], [19]. Together these processes drive a positive feedback in the erosion of landscape connectivity to the point that large patches also become subject to local extinction and the patch-area effect is lost again (Figure 1C). Ecological resilience is then impaired, and the system undergoes a regime-shift characterized by the loss of most habitat specialist species at both the patch and landscape scale, and the proliferation of generalist species that can successfully exploit edge and human-modified habitats [17], [20].

### Predictions for Biodiversity Patterns

Our model predicts a change in abundance (per unit of area), alpha diversity (species richness per unit of area in each patch) and gamma diversity (number of species across the entire landscape) of habitat specialist species as native vegetation cover is reduced at the landscape scale. Comparing across landscapes characterised by increasing levels of total native vegetation loss we can expect that: (1) positive patch-area effects on local abundance and alpha diversity should be evident only at intermediate levels of native vegetation cover, and (2) an abrupt drop in gamma diversity should be observed at low levels of native vegetation cover. High levels of native vegetation cover can be expected to support consistently high levels of species abundance and alpha diversity across individual patches, and thus a high level of total gamma diversity across the landscape (Figure 1A). By contrast a systematic reduction in species abundance and alpha diversity across all patches and a concomitant drop in gamma diversity should occur at low levels of native vegetation cover (Figure 1C). At intermediate levels of vegetation cover, although species abundance and alpha diversity should be positively correlated to patch size, gamma diversity should remain high given the retention of most species at least in larger patches (Figure 1B). For habitat generalists, which are not expected to depend exclusively on native vegetation, we expect either (1) no clear pattern of change in species abundance and diversity in response to changes in landscape vegetation cover or individual patch sizes, or (2) an increase in abundance across all patches in landscapes with low levels of native vegetation cover following the extinction of habitat specialists.

### Empirical Test of Predictions for Biodiversity Patterns

To test the predictions of our regime-shift model we employed a dataset describing the distribution of 39 non-volant small mammal species across three pairs of 10,000-ha fragmented and continuously-forested landscapes in the Atlantic Plateau of São Paulo, Brazil. The Atlantic forest is the second largest tropical forest in South America and is characterized by having both an extremely rich and endemic biota, while also being one the most imperilled tropical forest ecosystems in the world [34]. Forest cover has been reduced to less than 16% of its original extent, mostly distributed in patches smaller than 50 ha and closer than 250 m to the nearest edge [35]. Non-volant small mammals - the most diverse group of mammals in the Neotropics - are good indicators of anthropogenic alterations in the Atlantic forest, exhibiting distinct [36]–[38] and rapid [39] responses to forest fragmentation. They are also known to play key ecological roles as seed predators and dispersers [40], while some generalist species have an important impact on human health by acting as the main reservoirs of certain diseases [41] and as agricultural pests [42].

**Figure 2:**
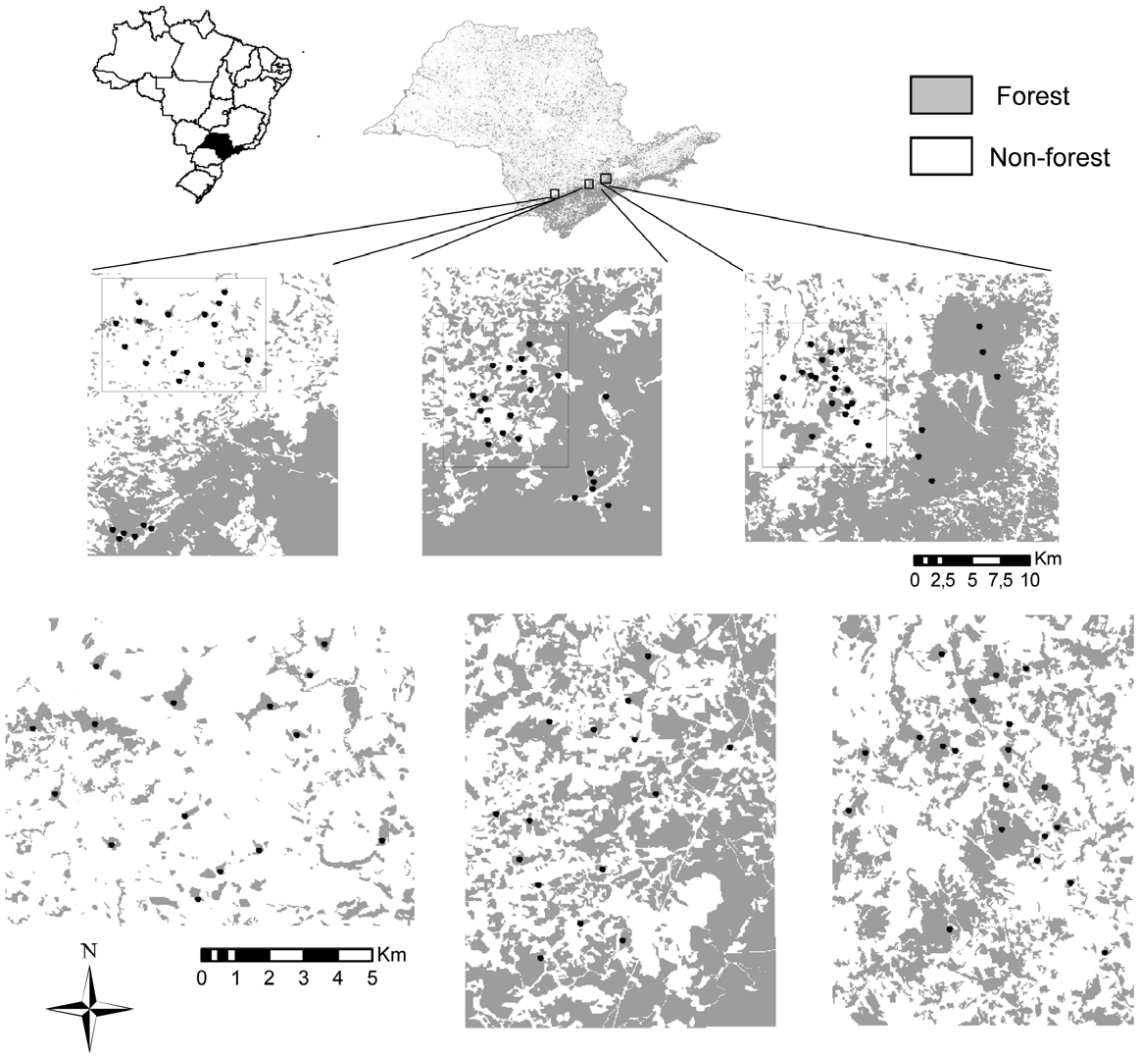
Distribution of Atlantic forest remnants and sampling sites in the fragmented and continuously-forested landscapes.

The three fragmented landscapes are characterised by different levels of remaining native forest (49, 31 and 11%, from now on referred to as 50, 30 and 10%), but are similar with respect to climate, topography, type of forest and of human use, and distance to areas of continuous forest (Figure S1 and S2, Table S1, and Text S1). The three paired continuously-forested landscapes are part of the Serra do Mar, the largest continuous tract of Atlantic forest remaining in Brazil [35]. Standardized, even-effort samples were taken from a set of forest patches of a comparable size-distribution in each fragmented landscape (15 to 20 per landscape, 50 in total), with six additional samples from each of the paired control areas (Figure 2). Size and shape of surveyed patches, distance to the nearest surveyed patch and distance of sampling sites to the nearest patch-edge did not differ among fragmented landscapes (Text S1).

This study design incorporates three features essential for testing the regime-shift model: (1) control of the patch-size distribution among landscapes, avoiding a bias towards larger remnants in more forested landscapes and permitting a comparable evaluation of patch-area effects among varying landscape contexts; (2) paired fragmented and continuously-forested landscapes within the same geographical range, controlling for biogeographic differences in assemblage structure as well as providing an appropriate reference point for measuring the conservation value of fragmented areas; and (3) sampling of landscape units large enough to encompass multiple sub-populations. Equally important, we explicitly incorporated species-specific differences in habitat requirement prior to attempting to disentangle the effects of landscape and patch-scale forest loss. This was achieved by grouping non-volant small mammals into forest specialist and generalist species based on previous independent work demonstrating inter-specific differences in habitat preference and specialization [37], [38] (Text S1).

We employed a standardised sampling protocol, using the same type, number and arrangement of traps in the 68 sites, and sampling each area for the same number of days, regardless of patch size. We used large pitfall traps, which are more effective than traditional live-traps, capturing a higher number of species and individuals, including rare species, and concentrated sampling effort in the wet season, when daily capture success is known to be higher [43]. At each site, we set a 100-m sequence of 11 pitfall traps (60-L buckets, 53.0 cm in depth and 40.0 cm in diameter), 10 m from each other and connected by a 50-cm high plastic fence. Four capture sessions of eight days each were conducted in each site, two per summer during two consecutive summers, totaling 32 days and 352 trap-nights in each site and 23,936 trap-nights in the 68 sites. In each session, all sites from a pair of fragmented and continuously-forested landscapes were sampled within one month. In one pair of fragmented and continuously-forested landscape, the capture sessions were conducted during the summers of 2001–2002 and 2002–2003 and, in the other two, during summers of 2005–2006 and 2006–2007. Animals were marked with numbered tags at first capture (Fish and small animal tag-size 1 – National Band and Tag Co., Newport, Kentucky). We collected voucher specimens of all species, which were determined by appropriate specialists (R. Rossi, A. Percequillo, Y. Leite, A.P. Carmignotto, J.A. de Oliveira, and C. Bonvicino), and are held at the Department of Zoology, University of São Paulo.

**Figure 3:**
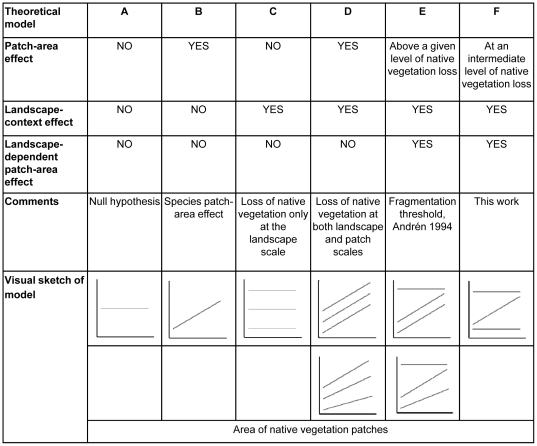
Candidate models representing species-patch area relationships in landscapes with different amounts of remaining native vegetation. Sketches represent the relationships between even-effort samples of total abundance or species richness (y axis) and patch area (x axis) in different landscapes (lines). By definition generalist species should not respond (A) or respond positively (C) to the loss of native vegetation. The simple patch-area effect model predicts that the abundance and richness of forest specialists increase with patch area, irrespective of landscape context (B). Alternatively, the amount of native vegetation at the landscape scale may determine the abundance and richness of forest specialists regardless of (C) or in combination with patch area (D). Finally, specialist species may respond to patch area only above a certain threshold of native vegetation loss at the landscape scale (E), or only at intermediate levels of native vegetation loss, above which individual patches (regardless of size) cannot support viable populations (F). For both D and E, the strength of the patch-area effect may depend upon landscape context.

We evaluated the data on the total number of individuals (abundance) and species (richness) of both specialist and generalist taxa in each of the three fragmented landscapes with eight alternative theoretical models (Figure 3), and employed an information-theoretic model selection approach to identify the most plausible candidates [44]. Candidate models encompassed a null hypothesis, a simple patch-area effect, a landscape-context effect, a combined landscape-context and patch-area effect, and two models that describe landscape-dependent patch-area effects: only in the two most deforested landscapes as proposed by Andrén [24], or only in the landscape with an intermediate level of deforestation, as proposed by our conceptual model (Figure 1 and 3).

All candidate models included combinations of linear and/or constant functions, which represent (1) the lack of relationship between the dependent variables and patch area in all landscapes, (2) a positive relationship with patch area regardless of landscape context, or (3) a positive relationship with patch area in one, two or three landscapes depending on landscape context (Text S1). The log-likelihood of each model was calculated as the sum of the log-likelihoods of their component functions. The maximum likelihood estimates for coefficients in each model were found with optimization routines [45] as the set of values that minimized the whole model negative log-likelihood (i.e. the sum of the negative log-likelihood of the component functions).

Species abundances were calculated as the sum of the number of individuals captured in each site and were modeled as a Negative Binomial variable in models that lack patch area effects (constant functions), and otherwise as a Poisson variable. Species richness was calculated as the average number of species between the two years of sampling to avoid overestimating the number of species present simultaneously in each patch, and was modeled as a Poisson variable in all cases. In order to express the expected value as the average number of species per year, we used as a response variable the mean number of species between years multiplied by two and included in the model an offset of two, which accounts for the two sampling years. Patch areas were converted by their logarithms (base 10). All analyses were conducted in the R environment, version 2.8.0 [46], and codes are available under request from the authors.

The Akaike Information Criterion corrected for small samples (AICc) was calculated for each model from their log-likelihoods, number of parameters and sample sizes, and the model with the lowest AICc was considered the most plausible. The plausibility of alternative models were estimated by the differences in their AICc values in relation to the AICc of the most plausible model (ΔAIC), where a value of ΔAIC <2 indicates equally plausible models. The Akaike weights (wi) express the relative likelihood of each model, in a scale of 0 to 1.

## Results

In the set of 68 sampling sites we captured 3653 individuals from 39 non-volant small mammal species, including 27 rodents and 12 marsupials. Among those, 2219 individuals were from 27 forest specialist species (Table S2) and 1434 individuals from 12 generalist species (Table S3).

**Figure 4:**
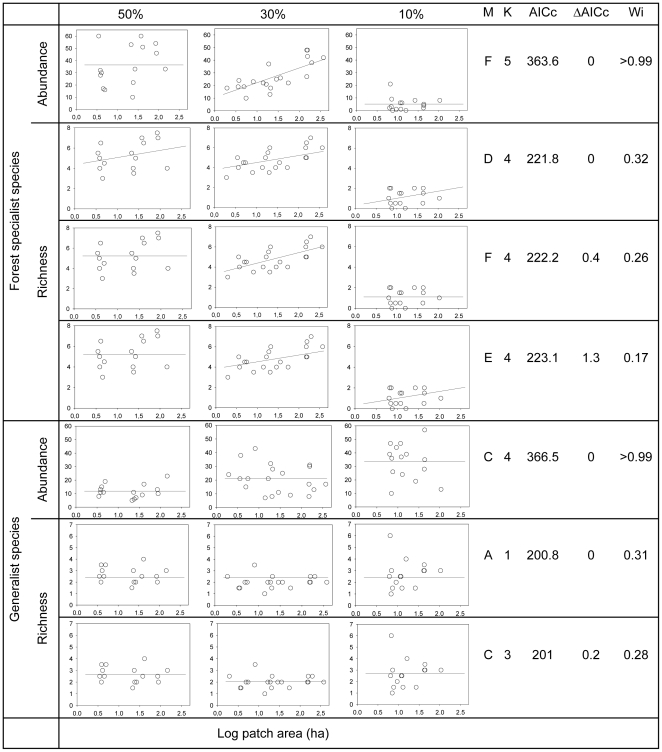
Plausible models describing the variation in non-volant small mammal abundance and richness in three Atlantic forest landscapes. The models describe the variation in even-effort samples of total abundance and species richness of specialist and generalist non-volant small mammals among forest patches in landscapes characterised by different levels of total forest loss. M - models (letters corresponding to models in Figure 3); K - number of parameters; AICc - Akaike Information Criterion for small samples; ΔAICc - difference between the AICc of a given model and that of the best model; Wi - Akaike weights (based on AIC corrected for small sample sizes).

As predicted by the regime-shift model, forest specialist species showed a strong landscape-dependent response to changes in patch area, with both total abundance and species richness being consistently affected by patch area only in the intermediate-forested (30%) landscape (Figure 4). For the abundance of forest specialist species, the landscape-dependent hypothesis of patch-area effects only in the landscape with intermediate forest cover was the only selected model and presented a high relative likelihood based on AIC_c_ model weights (w_i_>0.99), predicting a higher mean abundance in the two most forested landscapes, and a positive effect of patch area only in the 30% landscape (Figure 4, Table S4). For the richness of forest specialist species, although three models were equally plausible, all predicted a landscape-context effect, with mean richness being higher in the two most forested landscapes, and a strong patch-area effect only evident in the landscape with intermediate forest cover (30%), while no or only weak patch-area effects were predicted in the two landscapes with extreme (high or low) levels of forest cover (Figure 4, Table S4).

As expected for species that are not dependent on native forest, neither the total abundance nor richness of generalist species was affected by differences in patch size in any of the landscapes. Instead, the only selected model for the abundance of generalist species predicts only a landscape-context effect with mean abundance being higher in more deforested areas (w_i_ >0.99, Figure 4, Table S4). For the richness of generalist species, two models were equally plausible: the null model with no landscape-context or patch-area effect and one predicting a small landscape-context effect, with mean richness being lower in the landscape with 30% of remaining cover (Figure 4, Table S4).

**Figure 5:**
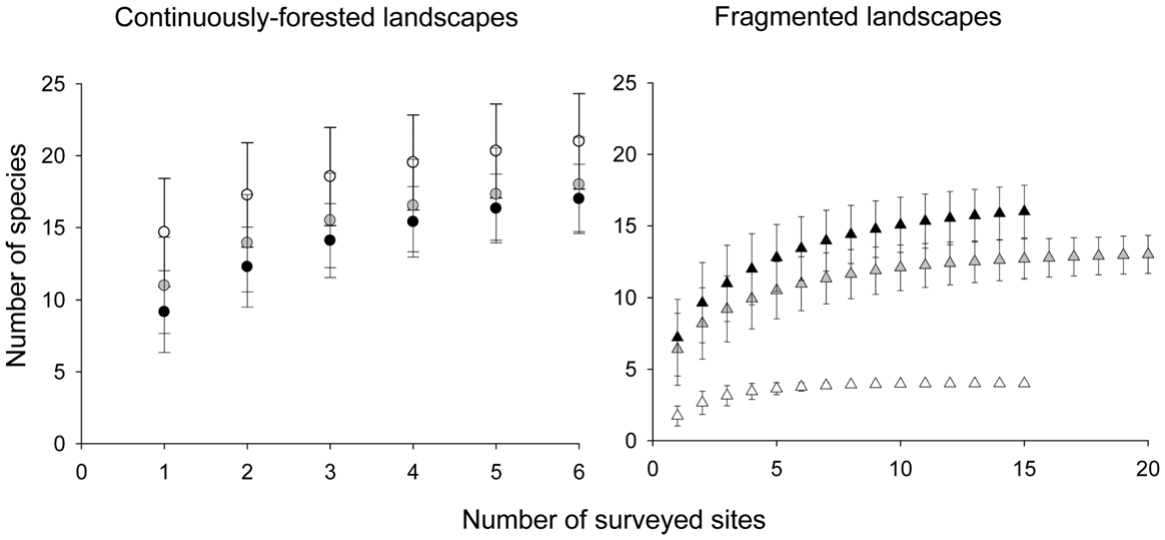
Species accumulation curves for each of the three pairs of continuously-forested and fragmented landscapes. Mean and 95% confidence intervals are presented. Color identifies the different pairs by the percentage of forest cover in the fragmented landscapes: black −50% forest cover; gray −30% forest cover; white −10% forest cover. Accumulation curves were generated in EstimateS 8.2.

In terms of the total number of forest specialist species found in each landscape (gamma diversity), both species-accumulation curves (Figure 5) and estimated richness (Table S2) indicate that the most deforested landscape (10% remaining cover) harboured 3–5 times less specialist taxa than the two most forested fragmented landscapes (30 and 50%) and control areas. As a result, while the Bray-Curtis similarity in species composition (presence/absence of forest specialist species) between the two most forested landscapes was 75.9%, falling well within the range of similarity between the control areas (73.7 to 82.1%), species composition in the most deforested landscape (10% forest cover) was comparatively less similar to that observed in the other fragmented landscapes (35.3 and 40%). Moreover, the two most forested fragmented landscapes accounted for 94 and 72% of the total number of specialist species found in the neighbouring control areas compared to 19% in the case of the most deforested landscape (Table S2). The strength of this observed shift in species composition is most clearly evident by the fact that all of the four most abundant specialist taxa in the two most forested landscapes (30 and 50%) were entirely absent from the landscape with only 10% remaining forest cover while common in the paired control area (Table S2).

## Discussion

We can draw two main conclusions from our results. First, the most plausible models included the effect of total landscape forest cover in all cases, with the effects observed in opposite directions for forest specialist and habitat generalist species. Second, the importance of patch-area effects varied between species groups and among landscapes as expected by the proposed conceptual model, with no patch-area effect on generalist species (irrespective of landscape context) and a strong and positive effect on the abundance and richness of forest specialist species in the landscape with intermediate forest cover. While the lack of evidence for a clear patch-area effect on the abundance and richness of forest specialist species in the most forested landscape agrees with Andrén's fragmentation threshold [24], [25], our finding of an analogous situation in the most heavily deforested landscape is novel. The observed reduction in abundance, local richness (alpha diversity) and landscape richness (gamma diversity) of forest specialist species, together with a proliferation of generalist taxa, provide strong support for the proposed regime-shift model, and the loss of ecological resilience in heavily deforested landscapes.

Our results also emphasize the critical importance of taking into account variation in species traits to evaluate biodiversity change across environmental and disturbance gradients. Specialist species - especially habitat specialists - have been shown to be particularly affected by environmental disturbance [47]. As such biodiversity indices that take into account the degree of specialization among species can often provide a more sensitive and robust method of measuring human impacts in natural systems [48], [49]. Geographical range size – one of the traits most often cited as a good predictor of extinction risk – is frequently correlated to niche breath [50], and has been commonly used as a proxy measure of the degree of habitat specialization among species [51]. Indeed endemic, specialized species tend to be not only more affected by human disturbances, but also less able to recover back to their initial state [50], [52]. Our species classification was based on known geographical distributions, and is supported by previous studies which have shown that among Atlantic forest, non-volant small mammals those with restricted distributions are also forest specialists and are largely absent from anthropogenic habitats [37], [38]. This pattern appears to be consistent among non-volant small mammal assemblages in other parts of the world [53].

Despite an increasing number of studies illustrating the importance of landscape-scale native vegetation cover on biodiversity [13], [15], [19], empirical tests of the potential for threshold effects have been limited to individual species [12], the interaction between total vegetation cover and configuration at the landscape scale [14], [19], or have been confounded by differences in patch-size distributions among landscapes [30]. Variable findings among previous studies have cast doubt on the relevance of native vegetation thresholds for biodiversity conservation [31]. Although we agree that the existence of a universal threshold value (e.g. 10–30% [24]) of native vegetation cover is highly unlikely, regime shifts such as that shown here, albeit with varying threshold values among different taxa and regions, maybe much more common than currently recognized. Once factors that are critical in determining variability in immigration rates are taken into account (i.e. differences in species dispersal ability, habitat specialization and the quality of the anthropogenic matrix), we believe that the model proposed here can provide a broadly applicable guide to understanding the consequences of native vegetation loss for biodiversity, and thus help develop more effective strategies for long-term conservation management in human-modified landscapes.

Although we only reported here the effects on biodiversity patterns, the observed abrupt change in biodiversity may have potentially serious ramifications for important biodiversity-mediated ecosystem services, such as the control of disease risk [54]. *Oligoryzomys nigripes* - the rodent responsible for the strong increase in the abundance of generalist species in the most degraded landscape (Table S3, abundance per patch 4.2±2.9 in the 50% landscape, 13.0±8.0 in the 30% landscape, and 27.3±12.0 in the 10% landscape) and previously known to be favoured by forest disturbance at smaller spatial scales [36], [37], [55], [56] - is the main reservoir of the hantavirus associated with the fatal hantavirus pulmonary syndrome in the Atlantic forest [41]. Not only are elevated abundances of the reservoir species known to increase transmission rates and pathogen reproduction success, but recent experimental work in Central America has also demonstrated that a reduction in non-volant small mammal diversity can have the effect of increasing hantavirus prevalence in the primary reservoir populations [57].

There are at least three main consequences of the proposed regime-shift model for conservation management. First, it re-emphasises the vital importance of developing conservation plans that account for landscape-scale processes [11], [13]. Second, it highlights the potential for maintaining high levels of biodiversity and ecological resilience in human-modified landscapes where a reasonable amount of native vegetation cover is retained [38]. Third, the model indicates that opportunities for enhancing biodiversity through local management and the restoration of native vegetation are greatest at intermediate levels of vegetation cover (Figure 1). In such intermediate landscapes immigration rates are reduced and biodiversity is concentrated in larger patches but the majority of the landscape-wide species pool is maintained, ensuring that the system still holds the potential to respond to local conservation interventions (e.g. restoration of smaller patches, or improvements to landscape connectivity through establishment of corridors or more structurally complex land-uses in the matrix) [16]. By contrast management effectiveness is likely to be lower in landscapes that have either high or low levels of native vegetation cover, either because biodiversity is already high across the entire landscape or because only a small fraction of the original species pool is available to support local recovery [16], [58]. Furthermore, our conceptual model and the predictions for changes to biodiversity supported by our data also suggest that the appearance of patch-area effects may represent an early warning indicator of an impending regime shift in the biodiversity of fragmented landscapes [6]. Given the time lag between landscape and biodiversity change [39], [59], however, opportunities for avoiding regime-shifts through management and restoration activities are likely to exist for a period of time after the initial human impact, and before extinctions occur.

Conservation activities are invariably constrained by limited resources [60], [61]. The proposed regime-shift model provides a framework for evaluating both existing biodiversity benefits and the likely effectiveness of management interventions, which should be taken into account for the efficient allocation of conservation and restoration resources [61]. This framework can help in developing more cost-effective and lasting conservation strategies by prioritizing areas for restoration in severely modified regions. At the same time it underlines the need for proactive measures elsewhere to prevent severe losses of vegetation that could incur either disproportionately high restoration costs or irreversible losses to biodiversity.

We believe that the conceptual model presented here will help advance our understanding of the ecological consequences of habitat loss and fragmentation by linking together three previously established ideas, namely: (1) the importance of patch size and isolation as determinants of local extinction risk [26]–[28], (2) the importance of structural thresholds in landscape pattern for determining dispersal success, extinction risk and biodiversity patterns [18], [19], [21], [22], [24], [25], and (3) the notion that ecological systems can present multiple stable-states and are thus vulnerable to regime shifts [3], [4], [8]. Future research should aim to test: (1) the predictions of the proposed regime-shift model for other taxonomic groups and ecological systems both through correlative studies in real landscapes and experimental work, (2) the main underlying mechanism that is assumed to drive the patterns observed in our data - that landscape-scale immigration rates are impaired by a landscape-wide loss of native vegetation cover, (3) the effectiveness of restoring native vegetation cover across different landscape contexts, and (4) the possible long-term consequences of such wholesale shifts in biodiversity for ecosystem functions and services.

## Supporting Information

Text S1 Description of the study region, mapping procedures and sampling design, details of data analysis, and additional references.
(0.08 MB DOC)

Table S1 Distribution of all forest patches in the three fragmented Atlantic forest landscapes with different proportions of forest cover.
(0.06 MB DOC)

Table S2 Number of individuals (and sites) where forest specialist, non-volant small mammals were sampled, total number of captured individuals, and observed and estimated richness in fragmented and continuously-forested landscapes.
(0.14 MB DOC)

Table S3 Number of individuals (and sites) where generalist, non-volant small mammals were sampled, total number of captured individuals, and observed richness in fragmented and continuously-forested landscapes.
(0.12 MB DOC)

Table S4 Mean (X) and standard deviation (SD) of non-volant small mammal richness and abundance among sampled sites in the three fragmented landscapes with different proportions of forest cover.
(0.07 MB DOC)

Figure S1 Distribution of land-use types in the three fragmented landscapes with different proportions of forest cover. A- Landscape with 50% forest cover in the municipalities of Piedade and TapiraÃ­; B- Landscape with 30% forest cover in the municipalities of Cotia and IbiÃ°na; and C- Landscape with 10% forest cover in the municipalities of RibeirÃ£o Grande and CapÃ£o Bonito.
(0.61 MB TIF)

Figure S2 Variation in vegetation structure among surveyed forest patches in the three fragmented landscapes. The graph represents a biplot of the first two axes of a Principal Component Analysis in a correlation matrix on the foliage density in five strata of the forest in the 50 surveyed patches. Color identifies the percentage of forest cover in the landscapes: black −50% forest cover; gray −30% forest cover; white −10% forest cover.
(0.03 MB TIF)
