# Supplementary material for: Beyond the Fragmentation Threshold Hypothesis: Regime Shifts in Biodiversity Across Fragmented Landscapes
Source: PLoS One. 2010 Oct 27;5(10):e13666. doi: 10.1371/journal.pone.0013666 (PMC2965145; doi:10.1371/journal.pone.0013666)
Supplement: Text S1 — Description of the study region, mapping procedures and sampling design, details of data analysis, and additional references. (0.08 MB DOC) [file pone.0013666.s001.doc]

**Text S1. Description of the study region, mapping procedures and sampling design, details of data analysis, and additional references.**

**General description of the study region**

We sampled six 10,000-ha landscapes located in three areas of the Atlantic plateau of São Paulo, encompassing parts of the municipalities of Piedade and Tapiraí, Cotia and Ibiúna, and Ribeirão Grande and Capão Bonito, in the State of São Paulo, Brazil. The whole region was once covered with Atlantic forest, classified as Lower Montane Atlantic Rain Forest [62], and in many areas is currently reduced to secondary forest fragments in different stages of regeneration. The altitude of the study area varies between 800-1,000 m above sea level [63]. Annual rainfall is between 1,220 and 1,810 mm and mean minimum and maximum temperatures are 17.3ºC and 28.4ºC for the warm-wet season (October to March) and 12.1ºC and 24.9ºC for the cool-dry season (April to September).

**Mapping procedures and sampling design**

In each of the three areas, two adjacent 10,000-ha landscapes were surveyed, one continuously-forested and one fragmented.

We used SPOT 5 satellite images (2005, resolution of 10 m) and intense ground-truthing to map land-use types in the three fragmented landscapes (Figure S1). In all of the fragmented landscapes, remaining forest patches are secondary regrowth in intermediate to advanced stages of regeneration, while the surrounding matrix is dominated by cattle pastures (48%, 44% and 50% of non-forested areas, for Piedade and Tapiraí, Cotia and Ibiúna, and Ribeirão Grande and Capão Bonito, respectively) and agriculture (26%, 20% and 35% of non-forested areas, for Piedade and Tapiraí, Cotia and Ibiúna, and Ribeirão Grande and Capão Bonito, respectively) (Figure S1). Although similar in terms of topography, relief, climate, type of forest, and type of human-use, the fragmented landscapes differ in the proportion of remaining native forest cover, varying from 49% (Piedade and Tapiraí), to 31% (Cotia and Ibiúna) and 11% (Ribeirão Grande and Capão Bonito) (Figure S1 and Table S1), and thus in the percentage of the landscape covered by the largest forest patch, the mean size of forest patches, and the mean distance of forest patches to their nearest neighbor (Table S1).

A total of 68 forest sites were sampled: 18 in continuously-forested landscapes (six per landscape), and 50 in fragmented landscapes - 15 in the landscape with 50% of forest cover, 20 in the landscape with 30%, and 15 in the landscape with 10%. In each fragmented landscape, sites were selected to (1) guarantee a minimum distance of 30 m from the edge in smaller patches (maximum possible distance in the smallest patches) and distances of more than 50 (and up to 200 m) whenever possible, (2) encompass a wide range of patch sizes, (3) maximize the overlap in the patch size distribution among landscapes, (4) maximize distance amongst surveyed patches, and (5) avoid spatial segregation among similar-sized patches.

The mean distance to the nearest surveyed patch was 1,462 m ± 714 m (maximum 3,737 m and minimum 423 m), and was not different among landscapes (ANOVA, F_3,50_ = 0.95, p = 0.392). Surveyed patches varied from 3 to 145 ha in the landscape with 50% of forest cover, from 2 to 374 ha in the landscape with 30%, and from 6 to 106 ha in the landscape with 10%. Both the size (ANOVA, F_3,50_ = 1.0, p = 0.561) and shape (ANOVA, F_3,50_ = 0.9, p = 0.495) of surveyed patches were similar among landscapes.

Distance of sites to the nearest patch-edge varied from 29 to 152 m in the landscape with 50% of forest cover, from 36 to 184 m in the landscape with 30%, and from 31 to 138 m in the landscape with 10%, and was similar among landscapes (ANOVA, F_3,50_ = 2.0, p = 0.146). Sampling sites within a given landscape are subject to varying edge effect intensity, although predictably this variation is tightly correlated with patch size, with samples in small patches being closer to edges (Pearson correlation r= 0.662, p< 0.001).

The percentage of forest in an 800-m circumference around sampling sites varied between 22 and 64% in the landscape with 50% of forest cover, 11 to 77 % in the landscape with 30%, and 5 to 41% in the landscape with 10%. Although sampling sites within a given landscape are surrounded by different amounts of forest, this variation is tightly correlated with patch size, with larger patches being surrounded by greater amounts of forest in all landscapes (Pearson correlation r= 0.825, p< 0.001).

Data collected after site selection indicated that sampled sites in different landscapes are similar with respect to vegetation structure (Figure S2), measured in terms of foliage stratification, which is a known indicator of forest disturbance and regeneration [36]. Studies on large mammals in the same sites also suggest that the three landscapes harbor similar assemblages of terrestrial large mammals, with top predators and large frugivores missing from all three areas, and mesopredators such as the common-opossum (*Didelphis aurita*) being consistently present in high numbers [64,65, G. Oliveira and R. Pardini unpublished data].

**Details of data analysis**

To account for the variation in habitat requirements among species, non-volant small mammals were grouped into forest specialists and generalists based on previous independent work demonstrating inter-specific differences in habitat preference [37,38]. These studies, conducted in fragmented Atlantic forest landscapes, showed that non-volant small mammal species that are negatively affected by the conversion of native forest into anthropogenic habitats, such as young regrowth, *Eucalyptus* plantations, cacao plantations, annual crops, and rural settlements, are those which are also restricted to forested biomes across their range (i.e. whose geographical distribution is restricted to the two Brazilian forest biomes – Amazon forest and Atlantic forest, and do not occupy the core areas of the open, savanna-like biomes – Caatinga and Cerrado, which separate the two forest biomes). In the absence of detailed natural history information such as diet or microhabitat use for the majority of the non-volant small mammals species captured in this study, forest specialists were considered as those known to be restricted to forested biomes (Atlantic forest and Amazonia), while generalists were classified as those that are known to also occupy open biomes adjacent to the Atlantic forest (Cerrado and Caatinga). Species distributions were based on the best available information and detailed maps of species geographical ranges [66,67,68].

Eight candidate models combining simple functions of the expected values of the response variables (species richness and abundance) were adjusted by maximum likelihood methods and compared using model selection:

Model A: the expected value of the response variable is a constant, and the model corresponds to the null hypothesis of no patch-area or landscape-context effect.

Model B: the expected values of the response variable are a linear function of patch area, and the model represents the simple patch-area effect hypothesis.

Model C: the expected values of the response variable are constants that vary among the three landscapes.

Model D: the expected values of the response variables are a linear function of patch area in each landscape, with similar or different slopes in the different landscapes.

Model E: the expected values of the response variables are constants in the landscape with 50% forest cover, but a linear function of patch area in the other two landscapes, with similar or different slopes between landscapes. This model represents the fragmentation threshold as proposed by Andrén [24].

Model F: the expected values of the response variables are constants in the landscape with 50% and 10% forest cover, with different values between these two landscapes, but a linear function of patch area in the landscape with 30% forest cover. This model represents the deforestation-driven regime-shift model proposed in this study.

Additionally, to check if the immediate landscape context around sampling sites could explain the observed patterns, we also ran a separate model where the expected values of the response variables (richness or abundance) were a positive linear function of the percentage of forest in an 800-m circumference surrounding sampling sites. This model never appeared among the selected models (wi < 0.096 for all dependent variables), demonstrating that differences in forest cover at smaller, sub-landscape spatial scales are insufficient to explain observed patterns.

**Additional references (not listed in the main text)**

62. Oliveira-Filho AT, Fontes MA (2000) Patterns of floristic differentiation among Atlantic Forests in Southeastern Brazil and the influence of climate. Biotropica 32: 793-810.

63. Ross JLS, Moroz IC (1997) Mapa Geomorfológico do Estado de São Paulo: escala 1:500.000. São Paulo: FFLCH-USP, IPT and FAPESP.

64. Negrão MFF (2003) Efeitos da fragmentação na comunidade de mamíferos médios e grandes na região de Caucaia, Mata Atlântica. MSc Dissertation, University of Brasilia, Brasília.

65. Espartosa KD (2009) Mamíferos terrestres de maior porte e a invasão de cães domésticos em remanescentes de uma paisagem fragmentada de Mata Atlântica: avaliação da eficiência de métodos de amostragem e da importância de múltiplos fatores sobre a distribuição das espécies. MSc Dissertation, University of São Paulo, São Paulo. Available: [http:\\www.teses.usp.br/teses/disponiveis/41/41134/tde-01052009-125425/](http://www.teses.usp.br/teses/disponiveis/41/41134/tde-01052009-125425/). Accessed December 2009.

66. Percequillo AR (2003) Sistemática das espécies sul-americanas do gênero *Oryzomys* Baird, 1858 Muroidea, Sigmodontinae. PhD Thesis, University of São Paulo, São Paulo.

67. Carmignotto AP (2004) Pequenos mamíferos terrestres do bioma Cerrado: padrões faunísticos locais e regionais. PhD Thesis, University of São Paulo, São Paulo.

68. Reis NR, Peracchi AL, Pedro WA, Lima IP (2006) Mamíferos do Brasil. Londrina: Universidade Estadual de Londrina. 437 p. Available: <http://www.uel.br/pos/biologicas/pages/arquivos/pdf/Livro-completo-Mamiferos-do-Brasil.pdf>. Accessed June 2010.
