# Supplementary material for: Beyond the Fragmentation Threshold Hypothesis: Regime Shifts in Biodiversity Across Fragmented Landscapes
Source: PLoS One. 2010 Oct 27;5(10):e13666. doi: 10.1371/journal.pone.0013666 (PMC2965145; doi:10.1371/journal.pone.0013666)
Supplement: Table S1 — Distribution of all forest patches in the three fragmented Atlantic forest landscapes with different proportions of forest cover. (0.06 MB DOC) [file pone.0013666.s002.doc]

**Table S1.** Distribution of all forest patches in the three fragmented Atlantic forest landscapes with different proportions of forest cover.

|  | **50%** | **30%** | **10%** |
| --- | --- | --- | --- |
| % of forest cover | 48.97 | 31.10 | 11.22 |
| % covered by the largest patch | 14.76 | 3.61 | 0.99 |
| Mean (± SD) size of patches (ha) | 15.37 ± 92.21 | 9.46 ± 29.66 | 3.75 ± 9.70 |
| Mean (± SD) distance to the nearest patch (m) | 52.73 ± 42.01 | 60.09 ± 58.52 | 101.46 ± 114.09 |
