# Supplementary material for: Beyond the Fragmentation Threshold Hypothesis: Regime Shifts in Biodiversity Across Fragmented Landscapes
Source: PLoS One. 2010 Oct 27;5(10):e13666. doi: 10.1371/journal.pone.0013666 (PMC2965145; doi:10.1371/journal.pone.0013666)
Supplement: Table S2 — Number of individuals (and sites) where forest specialist, non-volant small mammals were sampled, total number of captured individuals, and observed and estimated richness in fragmented and continuously-forested landscapes. (0.14 MB DOC) [file pone.0013666.s003.doc]

**Table S2.** Number of individuals (and sites) where forest specialist, non-volant small mammals were sampled, total number of captured individuals, and observed and estimated richness in fragmented and continuously-forested landscapes.

|  | **10%^a^** | **Con** | **30%^a^** | **Con** | **50%^a^** | **Con** |
| --- | --- | --- | --- | --- | --- | --- |
| *Marmosops incanus* |  | 25 (5) | 178 (19) | 76 (6) | 189 (15) | 31 (6) |
| *Brucepattersonius soricinus* |  | 53 (6) | 57 (13) | 6 (3) | 89 (14) | 15 (3) |
| *Monodelphis scalops/ M. americana* |  | 60 (6) | 52 (17) | 22 (6) | 88 (11) | 23 (5) |
| *Delomys sublineatus* |  | 90 (6) | 98 (18) | 53 (6) | 74 (13) | 15 (5) |
| *Sooretamys angouya* | 14 (5) | 8 (5) | 91 (19) | 15 (4) | 33 (11) | 3 (2) |
| *Thaptomys nigrita* |  | 101 (6) | 3 (3) | 29 (5) | 22 (9) | 11 (4) |
| *Gracilinanus microtarsus* | 42 (10) | 4 (3) | 30 (16) | 11 (5) | 12 (11) | 4 (2) |
| *Juliomys pictipes/ J. ossitenuis* | 12 (8) | 60 (6) | 4 (4) | 13 (6) | 10 (5) | 18 (5) |
| *Philander frenatus* |  | 3 (2) |  | 3 (2) | 7 (2) | 3 (2) |
| *Euryoryzomys russatus* |  | 21 (6) | 11 (5) | 52 (6) | 5 (4) | 29 (6) |
| *Monodelphis sorex* |  |  | 2 (2) |  | 5 (3) |  |
| *Monodelphis* sp.n. |  | 2 (1) |  | 5 (4) | 3 (3) | 1 (1) |
| *Phyllomys nigrispinus* |  | 1 (1) | 6 (6) | 5 (4) | 3 (3) | 1 (1) |
| *Akodon serrensis* |  | 59 (6) |  |  | 3 (2) |  |
| *Abrawayaomys ruschii* |  | 8 (5) |  |  | 1 (1) | 1 (1) |
| *Micoureus paraguayanus* | 8 (3) |  |  |  | 1 (1) |  |
| *Monodelphis ihering* |  | 8 (5) |  |  |  | 34 (6) |
| *Metachirus nudicaudatus* |  |  |  |  |  | 4 (2) |
| *Oecomys* gr. *catherinae* |  |  |  |  |  | 4 (1) |
| *Oxymycterus dasytrichus* |  |  | 10 (5) | 1 (1) |  | 3 (3) |
| *Rhipidomys mastacalis* |  | 1 (1) |  | 3 (3) |  |  |
| *Rhagomys rufescens* |  | 2 (2) | 1 (1) | 2 (2) |  |  |
| *Blarinomys brevicpes* |  |  |  | 1 (1) |  |  |
| *Marmosops paulensis* |  | 22 (5) |  | 1 (1) |  |  |
| Oryzomini sp.n. |  | 20 (6) |  | 1 (1) |  |  |
| *Delomys dorsalis* |  | 7 (4) |  |  |  |  |
| *Euryzygomatomys spinosus* |  | 1 (1) |  |  |  |  |
| **Number of individuals** | **76** | **556** | **543** | **299** | **545** | **200** |
| **Observed richness** | **4** | **21** | **13** | **18** | **16** | **17** |
| **Estimated richness (ACE)** | **4** | **23.0** | **13.6** | **21.0** | **17.1** | **18.3** |
| **Estimated richness (CHAO 1)** | **4** | **22** | **13** | **21** | **17** | **20** |
| **Estimated richness (JACKNIFE 1)** | **4** | **24.3** | **13.9** | **21.3** | **17.9** | **20.3** |
| **Estimated richness (MEAN)** | **4** | **23.1** | **13.5** | **21.1** | **17.3** | **19.5** |

Species-rank is ordered from highest to lowest abundance in the landscape with 50% forest cover.

^a^ Percentage of forest cover in fragmented landscapes.

Con, continuously-forested landscapes.
