# Supplementary material for: Beyond the Fragmentation Threshold Hypothesis: Regime Shifts in Biodiversity Across Fragmented Landscapes
Source: PLoS One. 2010 Oct 27;5(10):e13666. doi: 10.1371/journal.pone.0013666 (PMC2965145; doi:10.1371/journal.pone.0013666)
Supplement: Table S3 — Number of individuals (and sites) where generalist, non-volant small mammals were sampled, total number of captured individuals, and observed richness in fragmented and continuously-forested landscapes. (0.12 MB DOC) [file pone.0013666.s004.doc]

**Table S3. Number of individuals (and sites) where generalist, non-volant small mammals were sampled, total number of captured individuals, and observed richness in fragmented and continuously-forested landscapes.**

|  | **10%^a^** | **Con** | **30%^a^** | **Con** | **50%^a^** | **Con** |
| --- | --- | --- | --- | --- | --- | --- |
| *Akodon montensis* | 45 (12) | 115 (6) | 144 (20) | 35 (5) | 76 (15) | 2 (2) |
| *Oligoryzomys nigripes* | 409 (15) | 110 (6) | 259 (20) | 36 (6) | 63 (14) | 32 (6) |
| *Oligoryzomys flavescens* | 8 (5) | 2 (1) |  |  | 18 (10) |  |
| *Calomys tener* | 4 (4) |  | 9 (6) |  | 8 (7) |  |
| *Oxymycterus judex* | 6 (4) | 2 (2) |  |  | 7 (5) |  |
| *Necromys lasiurus* | 8 (2) |  | 2 (2) |  | 3 (2) |  |
| *Nectomys squamipes* |  | 1 (1) | 1 (1) | 1 (1) | 2 (2) |  |
| *Bibimys labiosus* |  |  | 2 (2) |  | 1 (1) |  |
| *Lutreolina crassicaudata* |  |  | 2 (2) |  |  |  |
| *Monodelphis kunsi* | 18 (11) |  |  |  |  |  |
| *Mus musculus* | 2 (1) |  |  |  |  |  |
| *Oxymycterus delator* | 1 (1) |  |  |  |  |  |
| **Number of individuals** | **501** | **230** | **419** | **72** | **178** | **34** |
| **Species richness** | **9** | **5** | **7** | **3** | **8** | **2** |

Species-rank is ordered from highest to lowest abundance in the landscape with 50% forest cover.

^a^ Percentage of forest cover in fragmented landscapes.

Con, continuously-forested landscapes.
