# Supplementary material for: Beyond the Fragmentation Threshold Hypothesis: Regime Shifts in Biodiversity Across Fragmented Landscapes
Source: PLoS One. 2010 Oct 27;5(10):e13666. doi: 10.1371/journal.pone.0013666 (PMC2965145; doi:10.1371/journal.pone.0013666)
Supplement: Table S4 — Mean (X) and standard deviation (SD) of non-volant small mammal richness and abundance among sampled sites in the three fragmented landscapes with different proportions of forest cover. (0.07 MB DOC) [file pone.0013666.s005.doc]

**Table S4.** Mean (*X*) and standard deviation (*SD*) of non-volant small mammal richness and abundance among sampled sites in the three fragmented landscapes with different proportions of forest cover.

|  | **50%** | | **30%** | | **10%** | |
| --- | --- | --- | --- | --- | --- | --- |
|  | ***X*** | ***SD*** | ***X*** | ***SD*** | ***X*** | ***SD*** |
| Specialist species abundance | 36.3 | 16.6 | 27.2 | 11.4 | 5.1 | 5.3 |
| Specialist species richness | 5.2 | 1.4 | 4.8 | 1.1 | 1.1 | 0.7 |
| Generalist species abundance | 11.9 | 5.0 | 21.0 | 10.4 | 33.4 | 13.3 |
| Generalist species richness | 2.6 | 0.7 | 2.0 | 0.5 | 2.7 | 1.2 |
