# Supplementary figures and images for: Beyond the Fragmentation Threshold Hypothesis: Regime Shifts in Biodiversity Across Fragmented Landscapes
Source: PLoS One. 2010 Oct 27;5(10):e13666. doi: 10.1371/journal.pone.0013666 (PMC2965145; doi:10.1371/journal.pone.0013666)

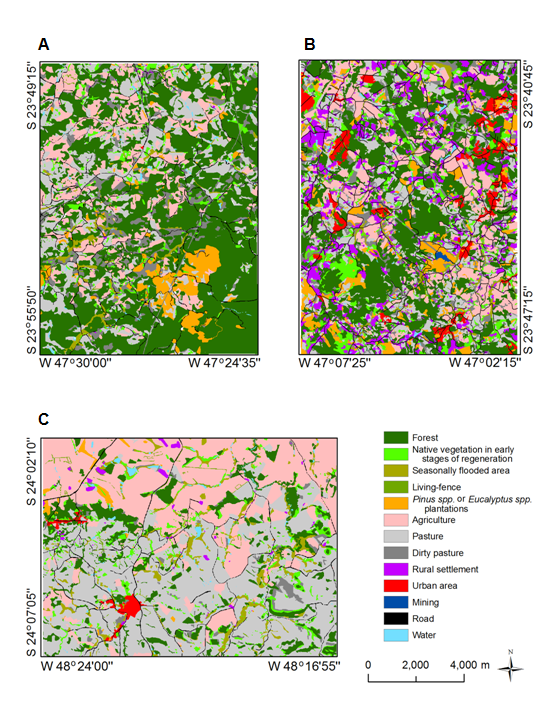

Supplement: Figure S1 — Distribution of land-use types in the three fragmented landscapes with different proportions of forest cover. A- Landscape with 50% forest cover in the municipalities of Piedade and TapiraÃ­; B- Landscape with 30% forest cover in the municipalities of Cotia and IbiÃ°na; and C- Landscape with 10% forest cover in the municipalities of RibeirÃ£o Grande and CapÃ£o Bonito. (0.61 MB TIF) [file pone.0013666.s006.tif]

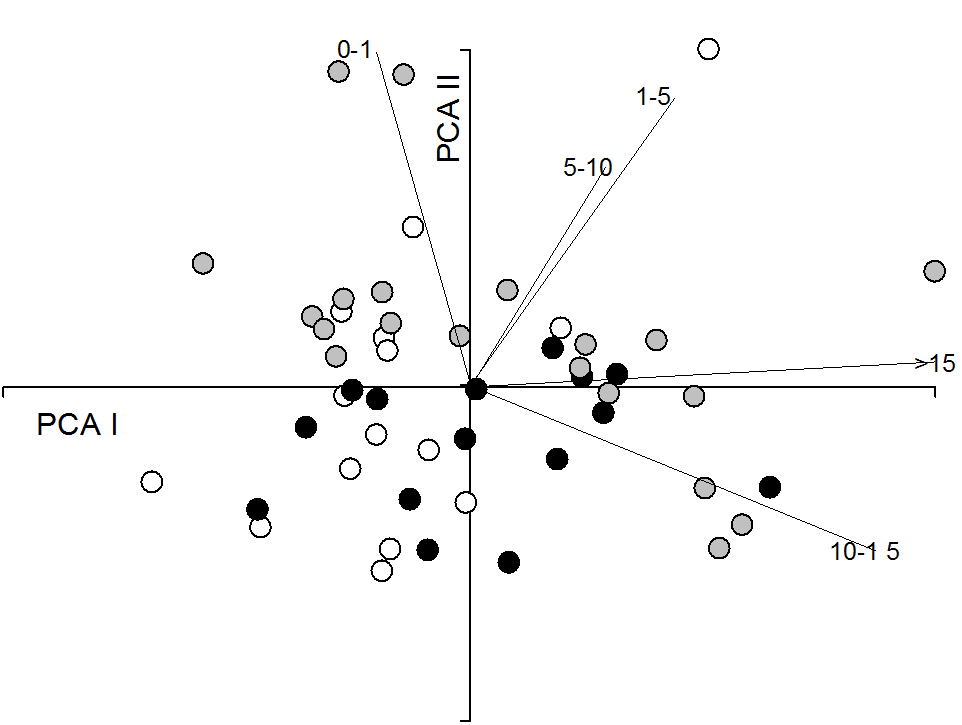

Supplement: Figure S2 — Variation in vegetation structure among surveyed forest patches in the three fragmented landscapes. The graph represents a biplot of the first two axes of a Principal Component Analysis in a correlation matrix on the foliage density in five strata of the forest in the 50 surveyed patches. Color identifies the percentage of forest cover in the landscapes: black −50% forest cover; gray −30% forest cover; white −10% forest cover. (0.03 MB TIF) [file pone.0013666.s007.tif]
